# Rest and Stress Left Atrial Dysfunction in Patients with Atrial Fibrillation

**DOI:** 10.3390/jcm12185893

**Published:** 2023-09-11

**Authors:** Angela Zagatina, Maria Rivadeneira Ruiz, Quirino Ciampi, Karina Wierzbowska-Drabik, Jaroslaw Kasprzak, Elena Kalinina, Irina Begidova, Jesus Peteiro, Rosina Arbucci, Sofia Marconi, Jorge Lowenstein, Alla Boshchenko, Fiore Manganelli, Jelena Čelutkienė, Doralisa Morrone, Elisa Merli, Federica Re, Clarissa Borguezan-Daros, Maciej Haberka, Ariel K. Saad, Ana Djordjevic-Dikic, Nithima Chaowalit Ratanasit, Fausto Rigo, Paolo Colonna, José Luis de Castro e Silva Pretto, Fabio Mori, Maria Grazia D’Alfonso, Miodrag Ostojic, Bojan Stanetic, Tamara Kovacevic Preradovic, Fabio Costantino, Andrea Barbieri, Rodolfo Citro, Annalisa Pitino, Mauro Pepi, Scipione Carerj, Patricia A. Pellikka, Eugenio Picano

**Affiliations:** 1 Cardiology Department, Research Scientific Cardiocenter “Medika”, 197110 St. Petersburg, Russia; zag_angel@yahoo.com (A.Z.); lennohka@mail.ru (E.K.); irina.begidova@mail.ru (I.B.); 2 Cardiology Department, University Hospital of Virgen Macarena, 41009 Seville, Spain; mariariva.1993@gmail.com; 3 Fatebenefratelli Hospital of Benevento, 82100 Benevento, Italy; qciampi@gmail.com; 4 Department of Internal Disease and Clinical Pharmacology, Medical University, 93-510 Lodz, Poland; wierzbowska@ptkardio.pl; 5 Cardiology Department, Bieganski Hospital, Medical University, 93-510 Lodz, Poland; kasprzak@ptkardio.pl; 6 CHUAC—Complexo Hospitalario Universitario A Coruna, University of A Coruna, 15071 La Coruna, Spain; jesus.peteiro.vazquez@sergas.es; 7 Cardiodiagnosticos, Investigaciones Medicas Center, Buenos Aires C1082, Argentina; rosinaarbucci@hotmail.com (R.A.); sofi_1151@hotmail.com (S.M.); lowensteinjorge@hotmail.com (J.L.); 8 Cardiology Research Institute, Tomsk National Research Medical Centre of the Russian Academy of Sciences, 634028 Tomsk, Russia; allabosh@mail.ru; 9 Cardiology Department, SG Moscati Hospital, 83100 Avellino, Italy; fiore.caranci@hotmail.it; 10 Centre of Cardiology and Angiology, Clinic of Cardiac and Vascular Diseases, Institute of Clinical Medicine, Faculty of Medicine, Vilnius University, Centre of Innovative Medicine, LT-10257 Vilnius, Lithuania; jelena.celutkiene@santa.lt; 11 Cardiothoracic Department, University of Pisa, 56126 Pisa, Italy; doralisamorrone@gmail.com; 12 Department of Cardiology, Ospedale per gli Infermi, Faenza, 48100 Ravenna, Italy; elisamerli@libero.it; 13 Department of Cardiology, Ospedale San Camillo, 00149 Roma, Italy; re.federica77@gmail.com; 14 Cardiology Division, Hospital San José, Criciuma 88801-250, Brazil; clarissabdaros@cardiol.br; 15 Department of Cardiology, SHS, Medical University of Silesia, 40-635 Katowice, Poland; mhaberka@op.pl; 16 División de Cardiología, Hospital de Clínicas José de San Martín, Buenos Aires C1120, Argentina; arielsaad@gmail.com; 17 Cardiology Clinic, University Center Serbia, Medical School, University of Belgrade, 11000 Belgrade, Serbia; skali.ana7@gmail.com; 18 Division of Cardiology, Department of Medicine, Siriraj Hospital, Mahidol University, Bangkok 10700, Thailand; nithima.cha@mahidol.ac.th; 19 Department of Cardiology, Dolo Hospital, 30031 Venice, Italy; faustorigo@alice.it; 20 Cardiology Division, Bari University Hospital, 70100 Bari, Italy; colonna@tiscali.it; 21 Hospital Sao Vicente de Paulo e Hospital de Cidade, Passo Fundo 99010-080, Brazil; jlpretto@cardiol.br; 22 SOD Diagnostica Cardiovascolare, DAI Cardio-Toraco-Vascolare, Azienda Ospedaliera-Universitaria Careggi, 50139 Firenze, Italy; morif@aou-careggi.toscana.it (F.M.); mariagrazia.dalfonso@gmail.com (M.G.D.); 23 Department of Noninvasive Cardiology, University Clinical Center, School of Medicine, 78000 Banja-Luka, Bosnia and Herzegovina; mostojic2011@gmail.com (M.O.); bojan.stanetic@gmail.com (B.S.); tamara.kovacevic@medicolaser.info (T.K.P.); 24 Cardiology Division, San Carlo Hospital, 85100 Potenza, Italy; marcofabiocostantino@tiscali.it; 25 Cardiology Division, Department of Biomedical, Metabolic and Neural Sciences, University of Modena and Reggio Emilia, Policlinico di Modena, 41124 Modena, Italy; barbieriandrea65@gmail.com; 26 Cardiology Division, Ospedale Ruggi di Aragona, 84100 Salerno, Italy; rodolfocitro@gmail.com; 27 CNR, Institute of Clinical Physiology, 56124 Pisa, Italy; annalisa.pitino@cnr.it; 28 Centro Cardiologico Monzino, IRCCS, 20138 Milano, Italy; mauro.pepi@cardiologicomonzino.it; 29 Cardiology Division, University Hospital Polyclinic G.Martino, University of Messina, 98166 Messina, Italy; scipione2@interfree.it; 30 Department of Cardiovascular Medicine, Mayo Clinic, Rochester, MN 55901, USA; pellikka.patricia@mayo.edu

**Keywords:** atrial fibrillation, left atrium, reservoir function, strain, stress echocardiography

## Abstract

Background: Left atrial (LA) myopathy with paroxysmal and permanent atrial fibrillation (AF) is frequent in chronic coronary syndromes (CCS) but sometimes occult at rest and elicited by stress. Aim: This study sought to assess LA volume and function at rest and during stress across the spectrum of AF. Methods: In a prospective, multicenter, observational study design, we enrolled 3042 patients [age = 64 ± 12; 63.8% male] with known or suspected CCS: 2749 were in sinus rhythm (SR, Group 1); 191 in SR with a history of paroxysmal AF (Group 2); and 102 were in permanent AF (Group 3). All patients underwent stress echocardiography (SE). We measured left atrial volume index (LAVI) in all patients and LA Strain reservoir phase (LASr) in a subset of 486 patients. Results: LAVI increased from Group 1 to 3, both at rest (Group 1 = 27.6 ± 12.2, Group 2 = 31.6 ± 12.9, Group 3 = 43.3 ± 19.7 mL/m^2^, *p* < 0.001) and at peak stress (Group 1 = 26.2 ± 12.0, Group 2 = 31.2 ± 12.2, Group 3 = 43.9 ± 19.4 mL/m^2^, *p* < 0.001). LASr progressively decreased from Group 1 to 3, both at rest (Group 1 = 26.0 ± 8.5%, Group 2 = 23.2 ± 11.2%, Group 3 = 8.5 ± 6.5%, *p* < 0.001) and at peak stress (Group 1 = 26.9 ± 10.1, Group 2 = 23.8 ± 11.0 Group 3 = 10.7 ± 8.1%, *p* < 0.001). Stress B-lines (≥2) were more frequent in AF (Group 1 = 29.7% vs. Group 2 = 35.5% vs. Group 3 = 57.4%, *p* < 0.001). Inducible ischemia was less frequent in SR (Group 1 = 16.1% vs. Group 2 = 24.7% vs. Group 3 = 24.5%, *p* = 0.001). Conclusions: In CCS, rest and stress LA dilation and reservoir dysfunction are often present in paroxysmal and, more so, in permanent AF and are associated with more frequent inducible ischemia and pulmonary congestion during stress.

## 1. Introduction

Atrial fibrillation (AF) is common in patients with chronic coronary syndromes (CCS) or heart failure (HF), and its development is associated with a worse prognosis [1]. Left atrial (LA) myopathy is frequent in patients with paroxysmal or permanent AF [2]. Transthoracic echocardiography (TTE) detects LA myopathy through the combined assessment of LA volume index (LAVI) with two-dimensional echocardiography (2DE) and LA strain of the reservoir phase (LASr, also known as peak atrial longitudinal strain) with speckle-tracking echocardiography (STE) [3,4]. In patients with sinus rhythm (SR), a reduction of LASr is a predictor of incident future AF, stronger than LAVI dilation [5,6,7,8]. In principle, subtle forms of LA dysfunction are detectable during stress as a reduced functional LA reserve. LA abnormalities occult at rest can be unmasked as a stress-induced marked LAVI dilation or blunted LASr increase [9]. The present study hypothesis was that LA volume and function abnormalities absent at rest can be best appreciated with stress echocardiography (SE), and—in some patients—abnormalities present at rest can be normalized by stress administration eliciting an atrial functional reserve not utilized at rest. In this hypothesis-driven analysis of prospectively acquired data from accredited laboratories contributing to a multicenter international SE 2030 study [10], we assessed LAVI and (in a subset) LASr at rest and during stress in patients with SR without a history of AF, with SR with history of paroxysmal AF, and with permanent AF. 

## 2. Methods

**Patients**. The initial population comprised 3214 patients prospectively enrolled at 40 cardiology institutions from 14 countries, with recruitment from 16 March 2016, to 16 March 2023 [10]. Of 3214 patients initially considered and present in the data bank, 32 (0.9%) were excluded for an unclear history of atrial fibrillation; 36 (1.1%) were excluded for inadequate imaging of rest LAVI; and 104 were excluded (3.2%) for missing LAVI data during stress. Of these 3042, LASr data at rest and peak stress were available in 486 patients. Indication for SE was the assessment of inducible ischemia in patients with known or suspected CCS. Exclusion criteria were a poor acoustic window at rest, clinically significant valvular or congenital heart disease, and prognostically relevant non-cardiac diseases (advanced cancer, end-stage renal disease, or severe obstructive pulmonary disease). All patients underwent transthoracic echocardiography (TTE) and SE with an assessment of LAVI (mandatory) and LASr (optional). The study protocol was reviewed and approved by the institutional ethics committees in its latest versions as a part of the more comprehensive SE 2020 study (148-Comitato Etico Lazio-1, 16 July 2016; Clinical trials.Gov Identifier NCT 030.49995) and SE 2030 study 291/294/295 Comitato Etico Lazio-1, 8 March 2021; Clinical trials.Gov Identifier NCT NCT050.81115) [10]. 

**TTE**. TTE was performed using commercially available ultrasound machines. All patients underwent TTE at rest, including ejection fraction (EF), and, in a subset of 1148 patients, global longitudinal strain (GLS). All measurements were taken by certified cardiologists according to the recommendations of the American Society of Echocardiography and the European Association of Cardiovascular Imaging [11].

**SE**. SE was performed according to current guidelines as previously detailed [12,13]. Wall motion score index (WMSI) was calculated in each patient at baseline and peak stress, in a four-point score ranging from 1 (normal) to 4 (dyskinetic), using a 17-segment model of the LV [14]. The ejection fraction was measured for apical biplane views with Simpson’s method. B-lines (also known as lung comets) were identified with lung ultrasound as vertical lines departing from the pleural line and synchronous with respiration. These were evaluated with a simplified four-site scan in the third intercostal space, from mid-axillary to anterior axillary to mid-clavicular line, each space scored from 0 (normal horizontal A-lines) to 10 (white lung), with a cumulative score per patient from 0 (normal) to 40 (severely abnormal). The procedure of acquisition was standardized between centers through a web-based learning module before starting data collection. All readers (one for each center) underwent quality control as previously described [15]. 

### LA Measurements 

LAVI was measured from apical four- and two-chamber views with the modified method of disks and normalized by the body surface area. LASr was measured by STE using frame rates from 40 to 80/s. The left atrial strain was calculated from either an apical four-chamber view (average from six LA segments) or combined four- and two-chamber views (average value from 12 LA segments) according to recommendations [16,17]. LASr was calculated from LV-end diastole, with R-wave as the zero-reference time point used as a surrogate of end-diastole, feasible also in AF, differently from the P wave convention. The first positive peak corresponds to the LA reservoir phase, and values are expressed as percentage points, algebraically positive. LAS-r corresponds to early diastole with maximum relaxation of its wall. LAS in the conduit phase and the atrial contraction phase were not included in the data set since LAS-r is more reproducible, easier to measure, and better represents the global function of the LA. Measurements were averaged over three cardiac cycles when the patient had SR and five cycles when there was AF.

Based on previously reported lower limits of reference values, we considered an abnormal LAVI value ≥ 34 mL/m^2^ and an abnormal LASr value < 24% [18]. Previous studies have shown that the normal stress response is characterized by a reduction or mild increase in LAVI during stress, with an increase of LASr [19,20,21]. 

**Statistical analysis**. All data are presented as mean ± SD, number (percentage), or median (interquartile range) as appropriate. Group differences were evaluated using the Student’s *t*-test for normally distributed continuous variables, the Mann-Whitney *U* test for skewed continuous variables, and the chi-square or Fisher’s exact tests for categorical variables. One-way analysis of variance or Kruskal-Wallis tests were used to compare >2 groups. When a significant difference was found, post hoc testing with Bonferroni comparisons for identified specific group differences was used. Paired Student’s *t*-tests or Wilcoxon tests were used to compare differences within groups. Pearson’s correlation coefficient was used to examine relationships between continuous variables. All analyses were two-sided. Statistical significance was set at *p* < 0.05. All statistical calculations were performed using SPSS for Windows, release 20.0 (Chicago, IL, USA).

## 3. Results

**Figure 1:**
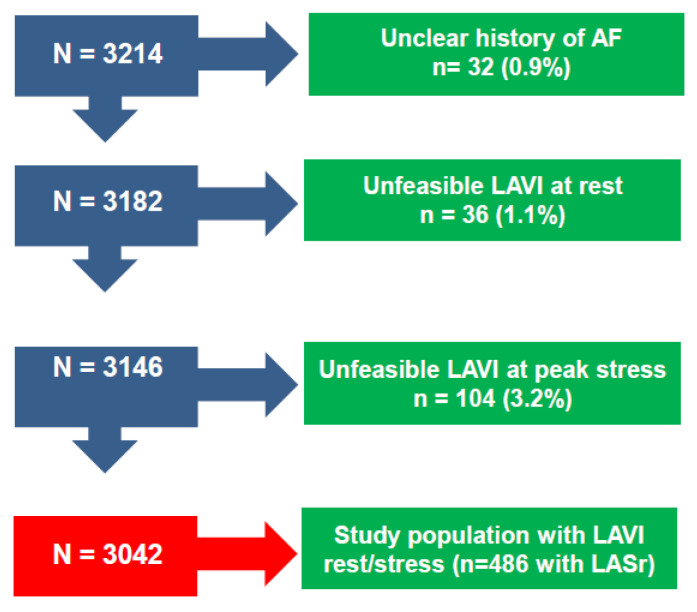
Consort Diagram Flow diagram showing how many individuals were excluded at each step (LAVI miss, LASr miss, unclear history of AF).

Accordingly, 3042 (1942 [63.8%] men; mean [±SD] age 64 ± 12 years) with interpretable LAVI formed the study group (Figure 1), with LASr data available in 486 (16.0%). The main clinical and resting echocardiographic features are reported in Table 1, with the overall population divided into SR without a history of AF (Group 1, n = 2749), SR with a history of paroxysmal AF (Group 2, n = 191), and permanent AF (Group 3, n = 102). We measured LAVI in all patients by selection and LASr in a subset of 486 (16.0%). 

**Resting TTE findings**. The resting echocardiographic LV EF in the entire study group was 59.9 ± 9.6%, with higher values in patients in SR than those with permanent AF (Table 1). A significant intergroup gradient was observed for LAVI (smallest in SR), LASr (highest in SR), and B-lines (lowest in SR). LAVI was abnormal in 754 patients (24.7%) and LASr in 241 (46.3%), with a progressive increase in the prevalence of abnormal results from Group 1 to 3 (Table 1).

**Figure 2:**
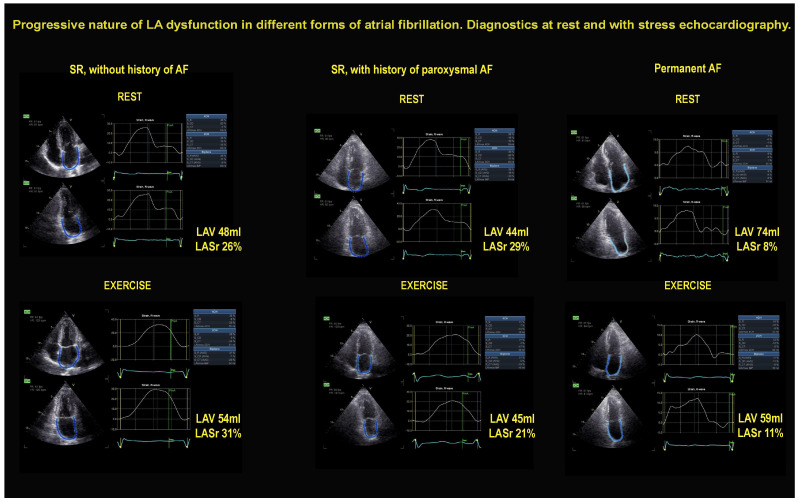
On the left, the normal response in a patient in SR showing a normal pattern, with small increase of LAV (48 mL rest, 54 mL stress) and increase of LASr (26% rest, 31% stress) during exercise. In the middle, a patient in SR but with a history of paroxysmal AF showing unchanged LAV (44 mL rest, 45 mL stress) and LASr decrease (29% rest, 21% stress) during exercise. On the right, a patient in permanent AF showing a near-normal LAV (74 mL rest, 59 mL stress) and severe reduction of LASr (8% rest, 11% stress) at rest and during exercise.

**SE findings**. The employed stresses were exercise (n = 1462; semisupine in 1250, peak or post-treadmill in 169, upright bicycle in 43), high-dose dobutamine (n = 417), or high-dose vasodilator (n = 1163; dipyridamole in 1137, adenosine in 26), based on the patient’s characteristics and laboratory expertise. The main SE findings are reported in Table 2. The Figure 2 shows an example of a patient from each of the three groups. A patient of Group 1 (left panel) shows a normal LA response during stress, with unchanged LAVI and LASr increase. A patient of Group 2 (middle panel) shows unchanged LAVI and LASr decrease during stress. A patient of Group 3 (right panel) shows a dilated LAV and reduced LASr, unchanged during stress. 

An abnormal stress response for B-lines and inducible RWMA were more frequent in patients of Group 3 than in those of Group 2 and Group 1 (Table 2). 

**Figure 3:**
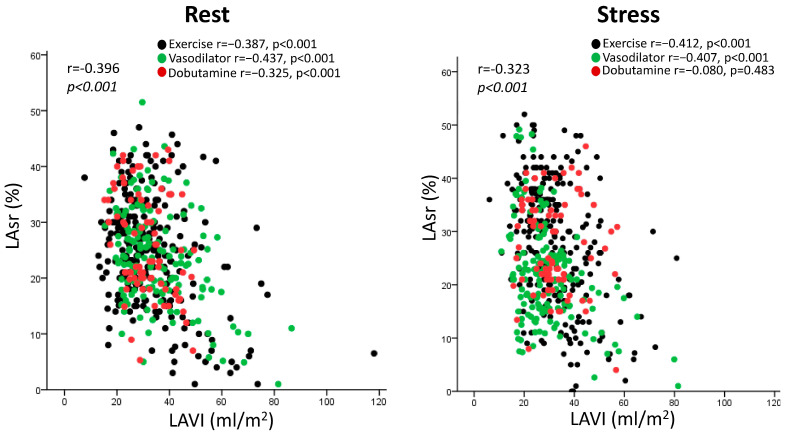
Scattergram of the patients with LAVI and LASr data at rest (**left panel**) and during stress (**right panel**), divided according to the stressors used (black: exercise; green: vasodilator; red: dobutamine).

Considering the subset of 486 patients with complete LAVI and LASr data, there was a linear inverse relationship between LAVI and LASr, both at rest and during stress, considering the different stressors (Figure 3). The correlation remained significant considering patients with exercise (n = 252, at rest, r = −0.387, *p* < 0.001, at peak stress: r = −412, *p* < 0.001) and pharmacological stress (n = 234, at rest: r = −0.409, *p* < 0.001, at peak stress: r = −0.269, *p* < 0.001). 

LAVI was normal at rest in 2290 patients and became abnormal during stress in 224 (9.8%). Of the 752 patients with abnormal LAVI at rest, 284 became normal during stress (37.8%), leading to a stress reclassification (from normal to abnormal, or vice-versa) in 508 patients (16.7%).

LASr was normal at rest in 253 patients and became abnormal during stress in 55 (21.7%). Of the 233 patients with abnormal LASr at rest, 71 became normal during stress (30.5%), leading to a stress reclassification (from normal to abnormal, or vice-versa) in 126 patients (25.9%).

## 4. Discussion

In this study, we assessed LAVI and (in a subset) LASr at rest and during stress in patients with CCS. We demonstrated that patients with AF had more frequent LA dilation and dysfunction at rest and during stress than patients with SR. Among patients with AF, LA dilation and dysfunction were more frequent and more severe in patients with permanent than in those with a history of paroxysmal AF but in SR at the time of testing. LA dysfunction was accompanied by more severe pulmonary congestion and regional wall motion abnormalities. SE led to a reclassification of LA (from normal to abnormal, or vice-versa) in 16.7% of patients by LAVI criteria and in 25.9% by LASr criteria.

### 4.1. Comparison with Previous Studies

Previous studies have shown that patients with AF exhibit LA dysfunction with increased LAVI and reduced LASr compared to controls without AF [22,23]. LA dilation is accompanied by LA dysfunction and a more profound functional impairment during stress, with more severe signs of pulmonary congestion [21]. Altogether, these data suggest that LA has an important impact on functional capacity, and early stages of LA dysfunction can be detected with a combination of LAVI and LASr. Multiple studies have analyzed the relevance of LAVI and LASr in AF patients, but fewer studies have assessed LAVI during stress, with a variety of different stressors combined with LASr with a multicenter design allowing enrollment of a large number of patients with different underlying comorbidities from different countries, who were evaluated with different stressors and involving different vendors. This heterogeneity is more likely to reflect real-world conditions and may increase the generalizability of the findings.

### 4.2. Clinical Implications

LAVI is now an integral part of the TTE data set in several conditions, from valvular heart disease to dilated or hypertrophic cardiomyopathy. These parameters can be obtained with little extra imaging and analysis time, not only at rest but also during stress in patients with CCS. LASr could be a valuable parameter added to volume data. LAVI and LASr provide a combined assessment of LA size and function, which are often uncoupled. LASr may help to identify an occult LA dysfunction not apparent at rest or reclassify as normal patients with alterations at rest. 

### 4.3. Study Limitations

LASr required more advanced technology than 2DE and was an optional variable, available only in 16% of patients. LA strain of the reservoir phase was available only in a minority of patients, limiting the robustness of this information. LAVI was assessed by 2DE but is more accurately measured with real-time three-dimensional echocardiography and artificial intelligence [24]. LASr is the easiest and most reproducible parameter to characterize LA function, but further information can be derived from the assessment of conduit and contraction phases, which may provide incremental value over LASr in predicting newly onset AF [25]. In addition, the multicenter nature of the study allowed a multi-vendor assessment of LA function. LASr may show some inter-vendor variability [26]. However, the adopted cutoff values for abnormality have been validated across different vendors, and the inter-vendor variability does not apply to stress-rest variation evaluated in the same patient with the same vendor. We focused on LASr, but right atrial strain can be even more important for predicting AF [27]. The study design was observational, and many clinical variables known to affect LAVi and LASr could not be controlled in the three study groups of this cohort. Patients with permanent AF were clearly different from those with paroxysmal AF and sinus rhythm for several traits, including older age, larger body surface area, higher prevalence of hypertension, and more frequent beta-blocker treatment. The study groups varied in size, and the size differences might have affected the conducted statistical analysis. Rather than the mean group size, the power to detect group differences will track more closely the harmonic mean of the group sizes (which usually implies lower-than-expected statistical power). The observational study design does not allow us to discern the contributions of these potential confounders from the direct, specific effect of arrhythmic history. The information on the duration of AF was not available in most patients since the structure of the data bank represents a trade-off between the priority of simplicity and the need for completeness. It is possible that the severity of LAVI and LASr abnormalities are related to the duration of arrhythmia history. Still, this aspect could not be addressed from the present data set. We did not include patients with persistent AF, likely to fall closer to permanent rather than paroxysmal AF, since the total atrial fibrillation burden is associated with progressive LA structural remodeling and a decrease in LA reservoir function [28,29]. We did not have information on antiarrhythmic drugs other than beta-blockers in our data bank, not specifically focused on AF. We did not have access to information on catheter ablation procedures and intraprocedural data, which are potentially important since a reduced LASr is a predictor of AF recurrence after ablation independently of LAVI and AF subtype [30].

### 4.4. Conclusions 

LA dilation and/or dysfunction are frequent in patients with AF and can be detected with resting TTE and SE with 2DE and STE. Both rest and stress LA abnormalities are more prevalent in patients with permanent than those with paroxysmal AF. However, patients with paroxysmal AF have more pronounced LA abnormalities than patients with SR. LA dysfunction is often accompanied by signs of pulmonary congestion during stress. Subtle forms of LA myopathy may be unmasked by SE in patients with paroxysmal or permanent AF.

## Figures and Tables

**Table 1:** Clinical and rest TTE findings in the study population.

	Overall (n = 3042)	SR (n = 2749)	Paroxysmal AF (n = 191)	Permanent AF (n = 102)	*p*-Value
Age	64 ± 12	63 ± 13 *^	69 ± 9 *	73 ± 8	<0.001
Male sex	1942 (63.8%)	1759 (64.0%)	118 (61.8%)	65 (63.7%)	0.828
BSA (m^2^)	1.89 ± 0.47	1.89 ± 0.49	1.91 ± 0.20	1.92 ± 0.20	0.048
Hypertension, n (%)	2391 (78.6%)	2125 (77.3%) *	174 (91.1%) *	92 (90.2%)	<0.001
Diabetes, n (%)	667 (21.9%)	599 (21.8%)	45 (23.6%)	23 (22.5%)	0.839
LBBB, n (%)	165 (5.5%)	147 (5.5%)	9 (4.9%)	9 (9.2%)	0.509
BB-therapy, n (%)	1658 (54.5%)	1422 (51.7%) *^	158 (82.7%) *	78 (76.5%)	<0.001
HR (bpm)	69.3 ± 12.2	69.0 ± 11.9 *	67.4 ± 11.4 *	80.5 ± 16.6	<0.001
SBP (mmHg)	130.2 ± 18.4	130.1 ± 18.3	131.5 ± 17.5	129.5 ± 20.5	0.564
DBP (mmHg)	78.0 ± 10.8	77.8 ± 10.7	79.5 ± 10.6	79.6 ± 13.7	0.033
Previous MI	799 (26.3%)	732 (26.6%)	43 (22.5%)	24 (23.5%)	0.374
PCI/CABG, n (%)	855 (28.1%) 200(6.6%)	786 (28.6%) 175 (6.4%)	44 (23.0%) 15 (7.9%)	25 (24.5%) 10 (9.8%)	0.453
LAVI (mL/m^2^)	28.3 ± 12.9	27.6 ± 12.2 *^	31.6 ± 12.9 *	43.3 ± 19.7	<0.001
LAVI > 34 (mL/m^2^)	752 (24.7%)	617 (22.4%) *^	70 (36.6%) *	65 (63.7%)	<0.001
LASr (%)	25.0 ± 9.4	26.0 ± 8.5 *^	23.2 ± 11.2 *	8.5 ± 6.5	<0.001
LASr < 24%	233 (47.9%)	181 (42.6%) *^	28 (71.8%) *	24 (100%)	<0.001
EF at rest (%)	59.9 ± 9.6	60.1 ± 9.6 *	61.2 ± 9.1 *	53.7 ± 11.1	<0.001
WMSI	1.12 ± 0.30	1.11 ± 0.29 *	1.12 ± 0.29 *	1.23 ± 0.42	0.001
B-lines ≥ 2	537 (18.6%)	454 (17.5%) *^	39 (20.4%) *	44 (43.6%)	<0.001
GLS (%)	−17.3 ± 4.1	−17.5 ± 3.9 *	−17.4 ± 3.9 *	−12.7 ± 4.5	<0.001
MR ≥ moderate	159 (7.4%)	127 (6.6%) *^	16 (11.0%) *	16 (20.3%)	<0.001
E/e’ (n = 1552)	10.8 ± 5.0	10.5 ± 4.8 *^	12.1 ± 5.7 *	15.5 ± 6.5	0.001
E/e’ > 15	224 (14.4%)	172 (12.5%) *	27 (22.9%) *	25 (43.9%)	<0.001
SPAP (n = 1349)	24.2 ± 10.6	23.7 ± 10.4 *	25.7 ± 9.3 *	32.2 ± 12.4	0.001

Abbreviations: AF: atrial fibrillation; BB: beta-blockers; BSA, body surface area; CABG: coronary artery by-pass grafting; DBP: diastolic blood pressure; EF: ejection fraction; GLS: global longitudinal strain; HR: heart rate, bpm: beats per minute; LASr, left atrial strain-reservoir phase; LAVI, left atrial volume index; LBBB: left bundle branch block; MI, myocardial infarction; MR, mitral regurgitation; PCI: percutaneous coronary intervention; SBP: systolic blood pressure; SPAP, systolic pulmonary artery pressure; SR: sinus rhythm; WMSI: wall motion score index; * *p* < 0.005 vs. Permanent AF, ^ *p* < 0.005 vs. Paroxysmal AF.

**Table 2:** SE findings in the study population.

	Overall (n = 3042)	SR (n = 2749)	Paroxysmal AF (n = 191)	Permanent AF (n = 102)	*p*-Value
Exercise	1462 (48.1%)	1305 (47.5%)	98 (51.3%)	59 (57.8%)	0.175
Dobutamine	417 (13.7%)	378 (13.8%)	29 (15.2%)	10 (9.8%)	
Vasodilator	1163 (38.2%)	1066 (38.8%)	64 (33.5%)	33 (32.4%)	
LAVI (mL/m^2^)	27.1 ± 12.8	26.2 ± 12.0 *^	31.2 ± 12.2 *	43.9 ± 19.4	<0.001
LAVI > 34 (mL/m^2^)	692 (22.7%)	555 (20.2%) *^	65 (34.0%) *	72 (70.6%)	<0.001
LAVI-dilators	385 (12.7%)	332 (12.1%) *^	29 (15.2%) *	24 (23.5%)	0.002
LASr (%)	25.8 ± 10.8	26.9 ± 10.1 *^	23.8 ± 11.0 *	10.7 ± 8.1	<0.001
LASr < 24%	217 (44.7%)	173 (40.9%) *^	23 (59.0%) *	21 (87.5%)	<0.001
dLASr (%)	7.23 ± 10.02	7.48 ± 9.77 *^	5.68 ± 9.61*	3.38 ± 15.12	<0.001
LASr-reducers	149 (30.7%)	125 (29.6%)*^	14 (35.6%)*	10 (41.7%)	0.353
EF (%)	67.6 ± 12.1	67.9 ± 11.9*	67.3 ± 11.6*	59.7 ± 15.5	<0.001
WMSI	1.14 ± 0.32	1.14 ± 0.31 *	1.17 ± 0.36 *	1.32 ± 0.53	<0.001
dWMSI	0.034 ± 0.22	0.030 ± 0.22 *	0.053 ± 0.18 *	0.083 ± 0.36	0.027
GLS (%)	−18.5 ± 4.8	−18.7 ± 4.7 *	−19.2 ± 4.1 *	−12.2 ± 4.7	<0.001
B-lines ≥ 2	885 (31%)	762 (29.7%) *	65 (35.5%) *	58 (57.4%)	<0.001
MR ≥ moderate	134 (7.6%)	111 (7.1%) *	10 (8.5%) *	13 (19.1%)	<0.001
E/e’ (n = 1432)	10.8 ± 4.7	10.4 ± 4.3 *^	12.5 ± 5.6 *	15.7 ± 6.1	0.001
E/e’ > 15	200 (14.0%)	144 (11.4%) *^	29 (25.4%) *	27 (49.1%)	<0.001
SPAP (n = 1116)	35.1 ± 16.8	34.3 ± 16.8 *	39.4 ± 16.1	46.1 ± 13.3	0.001

Abbreviations: as in Table 1. LAVI-dilators: an increase (stress > rest) ≥ 20% with absolute stress value > 34 mL/m^2^. LASr-reducers: a decrease (stress < rest) ≥ 20% with absolute stress value < 24%. EF reducers: stress < rest; GLS decreaser: stress < rest; WMSI-increaser: stress < rest for > 0.12. LAVI, EF, and WMSI, data were available in all patients (100%); B-lines in 2851 (93.7%); LASr in 486 patients (16.0%); GLS in 1148 (37.7%). * *p* < 0.005 vs. Permanent AF, ^ *p* < 0.005 vs. Paroxysmal AF.

## Data Availability

The main data underlying this article are available in the article and its online supplementary material. The raw data underlying this article cannot be shared publicly due to the privacy of individuals who participated in the study. These data will be shared on reasonable request to the corresponding author.

## Abbreviations

AF	atrial fibrillation
CCS	chronic coronary syndromes
EF	ejection fraction
HR	hazard ratio
LA	left atrial
LASr	left atrial strain
LAVI	left atrial volume index
LV	left ventricle
RWMA	regional wall motion abnormality
SE	stress echocardiography
SR	sinus rhythm
STE	speckle tracking echocardiography
TTE	transthoracic echocardiography
2DE	two-dimensional echocardiography
WMSI	wall motion score index

## Appendix A. Stress Echo 2030 Study Group

ARGENTINA: Cardiodiagnosticos, Investigaciones Medicas, Buenos Aires, Argentina: Jorge Lowenstein (lowensteinjorge@hotmail.com); Rosina Arbucci (rosinaarbucci@hotmail.com); Diego M. Lowenstein Haber (lowediego@hotmail.com); Sofia Marconi (sofi_1151@hotmail.com); Pablo M Merlo (pablommerlo@gmail.com)

Hospital Echocardiography Laboratory, Ramos Mejia Hospital, Buenos Aires, Argentina: Miguel Amor (miguelamor68@gmail.com); Hugo Mosto (hmosto@gmail.com); Michael Salamé (michael.f.salame@gmail.com); Patricia Carral (patriciabarral06@gmail.com); Germán Souto (germansouto87@gmail.com)

División de Cardiología, Hospital de Clínicas José de San Martín, Buenos Aires, Argentina: Ariel Saad (arielsaad@gmail.com)

BELGIUM: Department of Cardiology, Antwerp University Hospital, 2650 Edegem, Belgium: Caroline M. Van De Heyning (carovdh@msn.com)

BOSNIA AND HERZEGOVINA: Clinic of Cardiovascular Diseases, University of Banja Luka University Clinical Centre of the Republic of Srpska: Miodrag Ostojic (mostojic2011@gmail.com); Bojan Stanetic bojan.stanetic@gmail.com, Tamara Kovačević Preradović (tamara.kovacevic@medicolaser.info)

BRAZIL: Cardiology Division, Hospital San José, Criciuma, Brasil: Clarissa Borguezan-Daros (clarissabdaros@cardiol.br)

Hospital de Clinicas UFPR, Medicine Department, Federal University of Paranà, Curitiba, Brasil: Ana Cristina Camarozano (a.camarozano@yahoo.com.br)

BULGARIA: Heart and Brain Center of Excellence, University Hospital, Pleven, Bulgaria: Martina Samardjieva (martina_vl@abv.bg); Iana Simova (ianasimova@gmail.com)

CHINA: Department of Cardiovascular Ultrasound and Non-invasive Cardiology, Sichuan Provincial People’s Hospital, China: Zhang Hongmei (oiczhm@163.com); Yi Wang (wangyihdl@126.com); Ding Geqi (pldgq123@163.com); Key Laboratory of ultrasound in cardiac electrophysiology and biomechanics, the Affiliated Sichuan Provincial People’s Hospital of Electronic Science and Technology University of China, Chengdu, China: Zhang Qingfeng (qingfengzhang518@126.com)

Hebei, China: Yue Heng Wang (wyhucg@sina.com)

HUNGARY: Institute of Family Medicine, University of Szeged, Hungary: Albert Varga (varga.albert@med.u-szeged.hu); Gergely Agoston (drgergoagoston@gmail.com)

Second Department of Internal Medicine and Cardiology Center, University Hospital, Szeged, Hungary, and Elisabeth Hospital, Internal Medicine Department, Hódmezővásárhely, Hungary: Attila Palinkas (palinkasa@hotmail.com); Robert Sepp (sepprobert@gmail.com); Eszter D. Palinkas (palinkaseszti@hotmail.com)

ISRAEL: Chief of Echocardiography Unit, Soroka University Medical Center, Israele: Sergio Kobal (serkobal@clalit.org.il)

ITALY: Cardiology Division, Fatebenefratelli Hospital, Benevento, Italy: Quirino Ciampi (qciampi@gmail.com); Bruno Villari (brunovillari@gmail.com)

Cardiology Department, San Luca Hospital, Lucca, Italy: Lauro Cortigiani (lacortig@tin.it)

PO Umberto I°, Nocera Inferiore (ASL Salerno): Antonello D’Andrea (antonellodandrea@libero.it)

Cardiology Department, Parma University Hospital, Italy: Nicola Gaibazzi (ngaibazzi@gmail.com); Domenico Tuttolomondo (d.tuttolomondo@hotmail.it)

Department of Cardiology, Ospedale per gli Infermi, Faenza, Ravenna, Italy: Elisa Merli (elisamerli@libero.it)

Cardiothoracic Department, University of Pisa, Italy: Doralisa Morrone (doralisamorrone@gmail.com)

SOD Diagnostica Cardiovascolare, DAI Cardio-Toraco-Vascolare, e Cardiomyopathy unit, Azienda Ospedaliera-Universitaria Careggi, Italy: Fabio Mori (morif@aou-careggi.toscana.it); Maria Grazia D’Alfonso (mariagrazia.dalfonso@gmail.com); Iacopo Olivotto (iacopo.olivotto@unifi.it); Annamaria Del Franco (annamaria.delfranco@gmail.com);

Cardiology Department and Echocardiography Lab, University Hospital “San Giovanni di Dio e Ruggi d’Aragona,” Salerno, Italy: Rodolfo Citro (rodolfocitro@gmail.com)

Azienda Ospedaliera Rilevanza Nazionale A. Cardarelli Hospital, Naples, Italy: Rosangela Cocchia (rosangelacocchia@hotmail.com); Eduardo Bossone (ebossone@hotmail.com)

Villa Salus Foundation, IRCCS San Camillo Hospital, Venice, Italy: Fausto Rigo (faustorigo@alice.it)

ASST Santi Paolo e Carlo, Presidio Ospedale San Paolo, Milano: Francesca Bursi (francescabursi@gmail.com)

Ospedale San Camillo, Cardiology Division, Rome, Italy: Federica Re (re.federica77@gmail.com)

Cardiology Hospital, Policlinico University Hospital of Bari, Italy: Paolo Colonna (colonna@tiscali.it); Ilaria Dentamaro (ilaria.dentamaro@hotmail.it)

Cardiology Division, San Carlo Hospital, Potenza, Italy: Marco Fabio Costantino (marcofabiocostantino@tiscali.it)

Ospedale Moscati Avellino, Cardiology Division: Avellino, Italy: Fiorenzo Manganelli (fioreman@gmail.com)

LITHUANIA: Celutkiene Centre of Cardiology and Angiology, Clinic of Cardiac and Vascular Diseases, Faculty of Medicine, Institute of Clinical Medicine, Vilnius University, LT-03101 Vilnius, Lithuania: Jelena Celutkiene (Jelena.Celutkiene@santa.lt)

MEXICO: Instituto Nacional de Cardiologia Ignacio Chavez, Mexico City, Mexico: Hugo Rodriguez-Zanella (drzanella@gmail.com)

POLAND: Department of Internal Disease and Clinical Pharmacology, Lodz, Poland: Karina Wierzbowska-Drabik (wierzbowska@ptkardio.pl)

Chair of Cardiology, Bieganski Hospital, Medical University, Lodz, Poland: Jaroslaw D. Kasprzak (wierzbowska@ptkardio.pl)

University of Silesia, Cardiology Department, Katowice, Poland: Prof. Maciej Haberka (maciejhaberka@gmail.com)

RUSSIA: Cardiology Research Institute, Tomsk National Research Medical Centre of the Russian Academy of Sciences, Tomsk, Russia: Tamara Ryabova (rtr@cardio-tomsk.ru); Alexander Vrublevsky (avr@cardio-tomsk.ru); Alla Boshchenko (allabosh@mail.ru)

Department of Internal Medicine with a Course in Cardiology and Functional Diagnostics at the Medical Institute of the Peoples’ Friendship University of Russia, Moscow: Ayten Safarova (aytensaf@mail.ru); Tatiana Timofeeva (timtan@bk.ru)

Cardiology Department, Research Cardiology Center “Medika,” Saint Petersburg, Russian Federation: Angela Zagatina (zag_angel@yahoo.com)

SERBIA: Department of Noninvasive Cardiology, Institute for Cardiovascular Diseases, Dedinje, School of Medicine, Belgrade, Serbia: Aleksandra Nikolic (nikolicdrsasa@gmail.com);

Clinical Cardiology Department, Clinical Hospital Zvezdara, Medical School, University of Belgrade, Serbia: Milica Dekleva (dekleva.milica@gmail.com)

Cardiology Clinic, University Center Serbia, Medical School, University of Belgrade, Serbia: Ana Djordievic-Dikic (skali.ana7@gmail.com); Nikola Boskovic (belkan87@gmail.com); Vojislav Giga (voja2011@yahoo.com); Milorad Tesic (misa.tesic@gmail.com); Srdjan Dedic; Branko Beleslin (branko.beleslin@gmail.com)

SPAIN: CHUAC: Complexo Hospitalario Universitario A Coruna- University of A Coruna, La Coruna, Spain: Jesus Peteiro Vazquez (Jesus.Peteiro.Vazquez@sergas.es)

THAILANDIA: Division of Cardiology, Department of Medicine, Siriraj Hospital, Mahidol University, Bangkok, Thailand: Nithima Chaowalit Ratanasit (nithimac@hotmail.com)

USA: Department of Cardiovascular Medicine, Mayo Clinic, Rochester, Minnesota, USA: Patricia A Pellikka (pellikka.patricia@mayo.edu); Adelaide M. Arruda-Olson (ArrudaOlson.Adelaide@mayo.edu); Ratnasari Padang (Padang.Ratnasari@mayo.edu); Garvan C. Kane (kane.garvan@mayo.edu); Hector R. Villarraga (Villarraga.Hector@mayo.edu)

SIECVI-MAYO core team: Eugenio Picano (Chairman, eugeniopicanoofficial@yahoo.com); Patricia A. Pellikka (Co-chair, pellikka.patricia@mayo.edu); Quirino Ciampi (Principal Investigator, qciampi@gmail.com); Ylenia Bartolacelli (Paediatric Cardiology and Adult Congenital Heart Disease Unit, S. Orsola-Malpighi Hospital, Bologna, Italy ylenia.bartolacelli@gmail.com); Andrea Barbieri (REDCap for Data Archiving, barbieriandrea65@gmail.com); Giovanni Benfari (University of Verona, Verona, Italy: giovanni.benfari@gmail.com); Mauro Pepi (SIECVI President, Mauro.Pepi@cardiologicomonzino.it); Scipione Carerj (SIECVI President-elect scipione2@interfree.it).
